# Solubility and Stability of Some Pharmaceuticals in Natural Deep Eutectic Solvents-Based Formulations

**DOI:** 10.3390/molecules26092645

**Published:** 2021-04-30

**Authors:** Natali Rianika Mustafa, Vincent Simon Spelbos, Geert-Jan Witkamp, Robert Verpoorte, Young Hae Choi

**Affiliations:** 1 Natural Products Laboratory, Institute of Biology Leiden, Leiden University, 2333 Leiden, The Netherlands; verpoort@chem.leidenuniv.nl (R.V.); y.choi@chem.leidenuniv.nl (Y.H.C.); 2 Tiofarma, 3261 Oud-Beijerland, The Netherlands; 3 Division of Biological and Environmental Sciences and Engineering, King Abdullah University of Science and Technology, KAUST4700, Thuwal 23955, Saudi Arabia; geertjan.witkamp@kaust.edu.sa; 4 College of Pharmacy, Kyung Hee University, Seoul 02447, Korea

**Keywords:** natural deep eutectic solvents, pharmaceutical formulation, solubilization, stability, medicines

## Abstract

Some medicines are poorly soluble in water. For tube feeding and parenteral administration, liquid formulations are required. The discovery of natural deep eutectic solvents (NADES) opened the way to potential applications for liquid drug formulations. NADES consists of a mixture of two or more simple natural products such as sugars, amino acids, organic acids, choline/betaine, and poly-alcohols in certain molar ratios. A series of NADES with a water content of 0–30% (*w*/*w*) was screened for the ability to solubilize (in a stable way) some poorly water-soluble pharmaceuticals at a concentration of 5 mg/mL. The results showed that NADES selectively dissolved the tested drugs. Some mixtures of choline-based NADES, acid-neutral or sugars-based NADES could dissolve chloral hydrate (dissociated in water), ranitidine·HCl (polymorphism), and methylphenidate (water insoluble), at a concentration of up to 250 mg/mL, the highest concentration tested. Whereas a mixture of lactic-acid–propyleneglycol could dissolve spironolacton and trimethoprim at a concentration up to 50 and 100 mg/mL, respectively. The results showed that NADES are promising solvents for formulation of poorly water-soluble medicines for the development of parenteral and tube feeding administration of non-water-soluble medicines. The chemical stability and bioavailability of these drug in NADES needs further studies.

## 1. Introduction

Natural deep eutectic solvents, combinations of two or more simple natural products (individually solid at room temperature) such as sugars, polyalcohols, amino acids, organic acids, and bases in certain molar ratios, can form a liquid at room temperature, i.e., with a melting point much lower than those of its individual components. These might function as media for enzyme reactions and as solvent for water- and lipid-insoluble metabolites [1]. In any of these combinations, water might also be one of the ingredients [2]. In addition to the hypothetical media for bioprocessing, it seems that NADES have a great potential for applications in the field of natural products extraction, including medicines, cosmetics, and food additives, because they are non-toxic, they can be important solvents for extraction of many non-water-soluble compounds such as food flavors, fragrances, dyes, cosmetics, medicines, or agrochemicals; as reviewed in [3,4]. For example, NADES can be used for extractions of natural products, e.g., phenolics [5], natural colorants from safflower [6], anthocyanins from *Catharanthus roseus* flowers [7], vanillin from vanilla pods [8], and flavonoids from a medicinal plant *Scutellaria baicalensis* [9].

Prior to the introduction of NADES [1], synthetic ionic liquids (IL, defined as a salt or mixture of salts having a melting point below 100 °C) was a hot topic in green chemistry. These non-volatile solvents, that could be tailor made from synthetic acids and bases, were shown to be suitable solvents for chemical and enzymatic reactions, as well as for extractions [10]. Further developments resulted in the so-called deep eutectic solvents (DES) [11,12], which were mixtures of a hydrogen bond donor (HBD) and a hydrogen bond acceptor.

The ILs and DESs show great potential for various applications in the pharmaceutical field. Some ionic liquids were shown to have a strong activity against clinically important pathogens, such as rods, cocci, and fungi [13,14]. Activity against *Pseudomonas aeruginosa* and *Salmonella enterica* was found to be due to their physico-chemical properties that cause damage to biofilms [15]. Ionic liquids were found to be potential solvents for some pharmaceuticals [16,17]. The benzoylation of nucleosides was developed in ILs [18]. Paracetamol and ibuprofen could be dissolved in ILs [19] because of the hydrogen bond formation between the drug molecules with the cation- or anion part of the IL. In addition to being a solvent, the principle of ionic liquids was also proposed as an alternative form of an active pharmaceutical ingredient (API), as a way to avoid the problem of polymorphism and low solubility, which occurs with solid crystalline salts [20]. This affects the drug’s bioavailability and efficacy. Providing an API in IL form, referred to as “third generation of IL” [20], is considered to have several advantages [20,21]. Not only the physical and chemical properties, but the biological properties can also be tuned by choice of the counter ion [21]. A list of APIs that could be applied as ionic liquid were been reported by Ferraz et al. [10]. The potential of the API-ILs in drug delivery was discussed [22,23,24,25,26]. Furthermore, ionic liquids were recently developed as drug carriers to form self-assembling colloidal particles, where a small amount of IL (as API, or solvent to dissolve API) is dispersed in a large amount of oil, in the presence of a surfactant for a topical or transdermal [27] and oral applications [28].

Under conditions where a normal oral formulation of a medicine could not be administered, and either parenteral or tube feeding are the only ways, the medicine must be in a liquid form. However, the poor solubility of some APIs in water is a major problem. The IL and NADES might offer some new possibilities to solve this problem. Morrison et al. studied the thermal behavior of some deep eutectic solvents (choline chloride with either malonic acid or urea) and their potential for solubilizing drugs [29]. Manyfold increases were found if compared with solubility in water. Recently some publications reported the successful applications of NADES for improving solubilities of some medicines, e.g., salsalate [30], rutin [31], berberine [32], and resveratrol [33]. The term “therapeutic deep eutectic system” (THEDES) was introduced recently [34], where an API is one of the components of the deep eutectic system, making a delivery vehicle of the bioactive compound to improve bioavailability.

The aim of this study was to screen a series of NADES for solubilizing a series of well-known poorly soluble medicines. The solvents selected for this screening were known to be effective for extracting or dissolving various plant secondary metabolites. From our previous experience we knew that the NADES are quite selective, and thus to find a suitable solvent for any drug requires the testing of a large number of different NADES.

## 2. Results and Discussion

**Figure 1:**
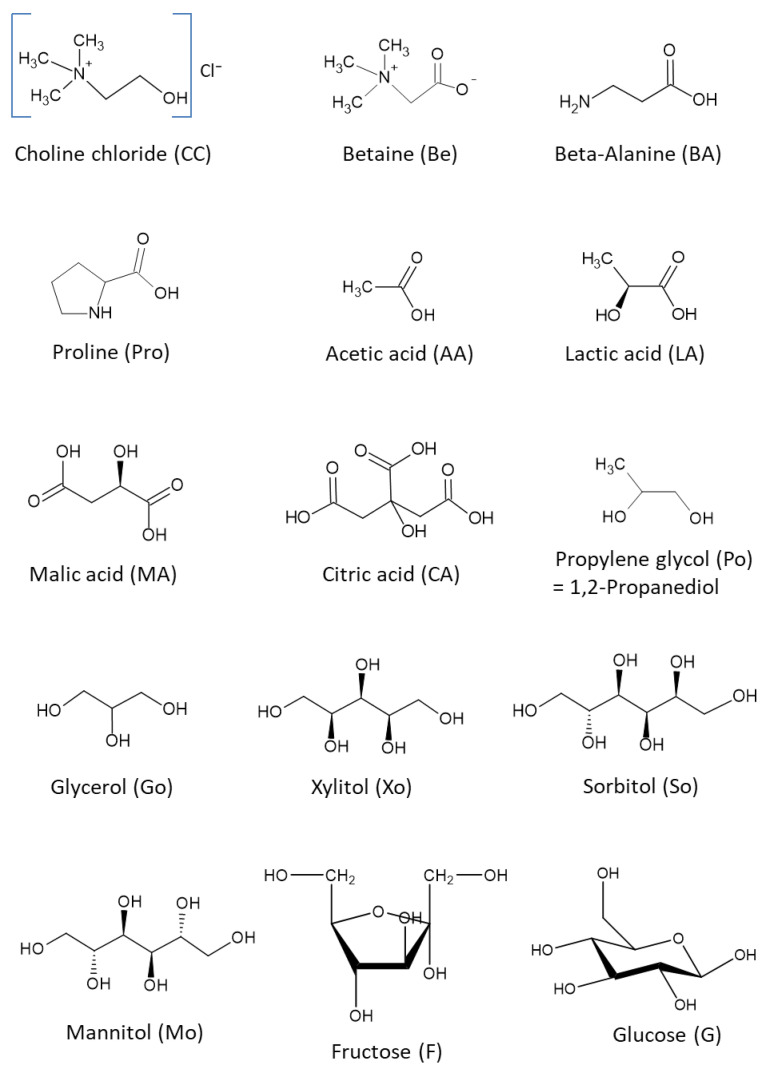
The chemical structures of some NADES components used in the study.

A series of different classes of NADES (acid–base, base–sugar, base–polyalcohol, acid–sugar, acid–polyalcohol, amino-acid–sugar, and sugar–sugar) was prepared for the solubility tests. The compositions (mole ratios) of the components in the NADES were based on our previous studies [1,2], where several different molar ratios of two or three components were tested. Compositions that provided clear liquids (NADES) were used in the present study. Some examples of NADES components are presented in Figure 1. Most of the NADES employed in this study include water as one of the ingredients, as the addition of water influences the solubility of compounds [4,5,35]. Moreover, the presence of a limited amount of water (<40% *w*/*w*) generally lowers the viscosity of NADES. The high viscosity is thought to be due to the extensive hydrogen bonds between NADES components, and additional water might break the hydrogen bonding between these components [35,36,37]. For example, addition of 5–20% (*w*/*w*) water to some viscous sugar-based NADES greatly decreases their viscosity. High viscosity affects homogenization/solubilization which, for example, can decrease the reaction rate in case of diffusion-controlled chemical reactions [38]. An increase of the amounts of water from 0.4% to 7.8% (*w*/*w*) in a low-melting mixture consisting of glucose, urea, and choline-chloride, decreased the viscosity by a factor of 50 [35,38]. A study of the role of inter-molecular and intra-molecular hydrogen bonding and energies in the formation of some choline-chloride based DESs was performed by Zhekenov et al. [12]. The results of the computer simulations showed that addition of water to a mixture of choline chloride and a hydrogen bond donor (HBD) such as urea, ethylene glycol, or glycerol, increased the number of hydrogen bonds between HBD–water, choline ion–water, chloride ion–water, and decreased those between HBD–choline ion, HBD–chloride ion, and HBD–HBD.

**Figure 2:**
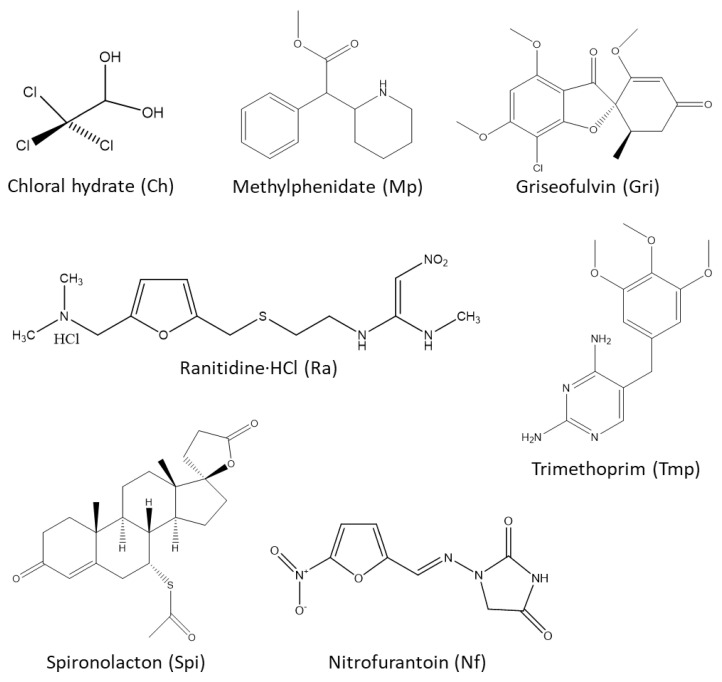
The chemical structures of the drugs tested in this study.

Several pharmaceuticals known for low solubility to non-water-soluble compounds (nitrofurantoin, trimethoprim, griseofulvin, methylphenidate, spironolactone) [39], or well-soluble but instable in water (trichloroacetaldehyde monohydrate or chloral hydrate); or polymorphic API (e.g., ranitidine·HCl) [20,40] were used in this study (Figure 2). Information about pKa and solubility of the APIs and some NADES components are presented in Table 1.

**Figure 3:**
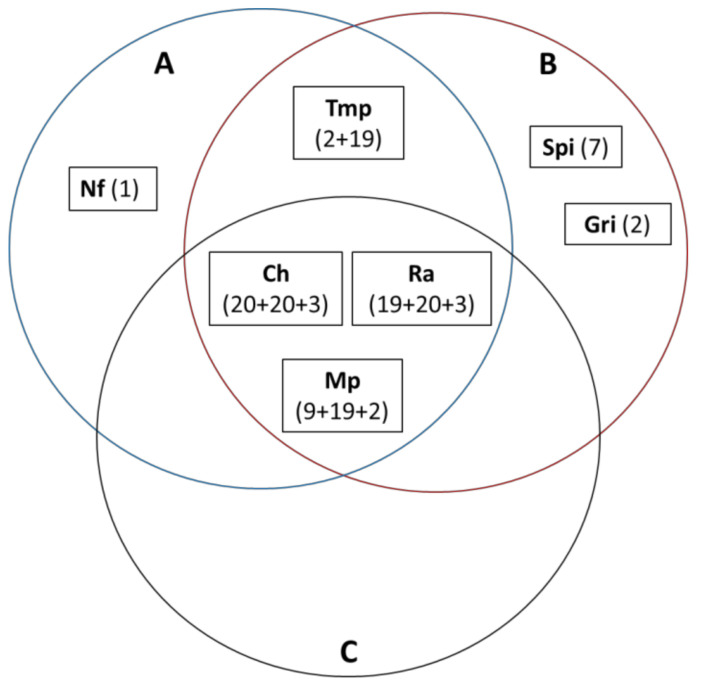
A Venn diagram showing the ability of three different groups of NADES to dissolve the pharmaceuticals (5 mg/mL). **A**—choline-chloride- or betaine-based NADES (Table 2), **B**—organic-acid-based NADES (Table 3), **C**—sugars based NADES (Table 4). Ch: chloral hydrate, Gri: griseofulvin, Nf: nitrofurantoin, Ra: ranitidine·HCl, Spi: spironolacton, Tmp: trimethoprim, and Mp: methylphenidate. A number or summed numbers in a bracket show the number of NADES of groups **A** + **B** + **C**, which dissolved the pharmaceutical.

The capability of three different groups of NADES to dissolve the pharmaceuticals is shown in a Venn diagram (Figure 3).

### 2.1. Solubility of the Pharmaceuticals in Choline Chloride- or Betaine-Based NADES

Choline chloride is one of the most common ingredients for DES. It is used in combination with urea [12], organic acids [34,41,42], sugars [42], ethylene glycol [12], or glycerol [12]. It is difficult to predict/anticipate the formation of a co-crystal or eutectic solvent only based on the knowledge of the molecular components, thus, so far there are no basic rules to predict what compounds can form eutectic mixtures/solvent [43]. With regards to eutectic liquid formation, both functional groups and stereo-chemical fit of the components are important factors that play a role in obtaining ILs and NADES. We observed that choline chloride with alcohols such as the sugars (fructose and sucrose) and sugar alcohols (glycol, propylene glycol, or xylitol), or in combination with an amino acid (e.g., proline), form stable NADES. Only certain molar ratios of two or more components provide the supramolecular assembly resulting in stable NADES. For example, a molar ratio of choline chloride:xylose:water (CCXW) of 5:2:3 failed to mix stoichiometrically, whereas the composition of 2:1:2 (Table 2) successfully formed a stable NADES. Additionally, when betaine·HCl was used instead of betaine, in a combination with a neutral compound (e.g., glucose, sucrose, sorbitol) or an acid (e.g., malic acid), it did not turn into a liquid.

The solubility data in choline-chloride- and betaine-based NADES are shown in Table 2. Of the tested medicines, chloral hydrate and ranitidine·HCl showed higher solubility than the other compounds in all tested NADES (Table 2, Table 3 and Table 4). It might be that both compounds have more or stronger hydrogen donor and acceptor groups that can easily interact with those in the NADES, if compared with the other pharmaceuticals tested.

Methylphenidate dissolves well in some choline-chloride-based ionic liquids with citric acid, maleic acid, malic acid, or lactic acid as the anion, which is related to the fact that methylphenidate can form a salt with any anion. However, if an amino acid (proline) was added into an ionic liquid of, e.g., choline-chloride–malic-acid at a certain ratio, apparently, the solubility of methylphenidate decreased, but this was not the case if the basic component betaine was added. It was the other way around if acetic acid was used instead of malic acid, Table 2.

A change in the NADES e.g., by addition of another “new” component or changing the components ratio, changes the molecular organization and thus possibly also the solubility of the drug. The supramolecular assembly and thus the solubility of compounds in a deep eutectic solvent is dependent on a balanced network of hydrogen bonds involving the solute molecules and the NADES molecules. Methylphenidate did not dissolve in most basic–neutral combinations, except for choline-chloride–propylene-glycol and betaine–sucrose. Trimethoprim, with two amino-groups, dissolves only in betaine-based ionic liquids, such as the mixtures of betaine–malic-acid–water (BeMAW) and betaine–malic-acid–proline–water (BeMAProW). However, it did not dissolve when malic acid was replaced by acetic acid (BeAAProW) or when proline was changed with glucose (in BeMAGW). From all NADES tested, nitrofurantoin could only be dissolved in choline-chloride–acetic-acid–proline–water (CCAAProW). There was no clear transparent solution formed by either griseofulvin or spironolactone (both are neutral compounds) with any basic NADES tested.

### 2.2. Solubility of the Pharmaceuticals in the NADES Based Organic Acid and Base

Table 3 shows the solubility of the test compounds in twenty-one other NADES, representing combinations of an organic acid with a sugar, an alcohol, or an amino acid. We observed that citric acid or maleic acid if combined with mannitol and water in a molar ratio of 1:1:7, failed to form NADES. However, if it was combined with sorbitol and water in a molar ratio of 1:1:7 (see Table 3), a transparent stable liquid was successfully formed. Mannitol and sorbitol are isomers, the difference is only in the stereochemistry of a hydroxyl group (Figure 1). Chloral hydrate is soluble in almost all acid-based NADES, except the mixture of citric-acid–proline–water (CAProW). The latter, interestingly, changed the transparent crystals of chloral hydrate to white insoluble crystals. Ranitidine·HCl was soluble in all acid-based NADES tested, except for the combination of acetic-acid–fructose-water (AAFW); it recrystallized after some hours of solubilization. Methylphenidate and trimethoprim tend to follow a similar trend. However, methylphenidate crystalized a few days after solubilization in the mixtures of malic-acid–glucose–water (MAGW) and citric-acid–proline–water (CAProW). Trimethoprim was also recrystallized in malic-acid–glucose–water (MAGW) and was not soluble at all in a combination of lactic-acid–beta-alanine–water (LABAW).

**Table 3:** Solubility of pharmaceuticals in the organic-acid-based NADES.

NADES	Molar Ratio	Pharmaceuticals						
		Ch	Gri	Mp	Nf	Ra	Spi	Tmp
CA:F:W	1:1:5	++	−	++	−	++	−	++
CA:S:W	1:1:6	++	−	+	−	+	−	+
CA:Go:W	1:1:2	++	−	+	−	++	−	+
CA:So:W	1:1:7	++	−	++	−	++	−	+
CA:Xo:W	1:1:5	++	−	++	−	++	−	++
CA:Po:W	1:1:3.7	++	−	++	−	++	+/P	++
CA:Pro:W	1:1:5	−	−	+/P	−	++	−	++
MA:F:W	1:1:7	++	−	++	−	++	−	++
MA:G:W	1:1:7	++	−	+/P	−	++	−	+/P
MA:F:G:W	1:1:1:7	++	−	++	−	++	−	++
MA:S:W	1:1:7	++	−	+	−	++	−	+
MA:Xo:W	1:1:4	++	−	++	−	++	−	++
MA:Po:W	1:1:3	++	−	++	−	++	++	++
MA:BA:W	1:1:3	++	−	+	−	++	−	++
MA:Pro:W	1:1:3.5	++	−	+	−	++	++	++
LA:F	5:1	++	−	++	−	++	++	+
LA:Po	1:1	++	−	++	−	++	++	++
LA:BA:W	2:1:1	++	−	++	−	++	−	−
AA:F:W	5:2:5	++	+/P	++	−	+/P	++	++
AA:Po	1:1	++	+	++	−	++	++	++
AA:BA	5:1	++	+	++	−	++	++	++

The concentration of the tested pharmaceuticals was 5 mg/mL at 25 °C. The solubility was measured by visual inspection. ++: clear solution within 30 min by vortexing and 25 min ultrasonication, +: clear solution in 24 h with vortexing, ultrasonication, and magnetic stirring, +/P: soluble within 30 min and precipitated within 24 h, (−): no solubility. AA: Acetic acid; BA: beta-Alanine, CA: citric acid monohydrate, MA: malic acid, LA: l-lactic acid, F: d-fructose, G: d-glucose monohydrate, S: sucrose, Go: glycerol, So: d-sorbitol, Xo: xylitol, Po: propylene glycol, Pro: l-proline, W: water, Ch: chloral hydrate, Gri: griseofulvin, Nf: nitrofurantoin, Ra: ranitidine·HCl, Spi: spironolacton, Tmp: trimethoprim, and Mp: methylphenidate.

Methylphenidate and trimethoprim dissolved slowly in some NADES, such as malic-acid–sucrose–water (MASW) and some citric-acid-based NADES (CASW, CAGoW, and CASoW). The viscosities of such NADES are relatively high if compared with the other NADES, which might cause a much longer dissolution time for the compounds. Interestingly, spironolacton was soluble only in some acid-based NADES such as LAF, LAPo, AAPo, AABA (without water), and also in some acid-based NADES containing water (MAPoW, MAProW, and AAFW). Apparently acetic acid and proline are important components in the NADES to dissolve this compound, which lacks a hydrogen bond donor. Griseofulvine also lacks an HBD function and requires acetic acid for solubilization (AAPo and AABA). None of the NADES of this class dissolved nitrofurantoin (Table 3).

The results in Table 2 and Table 3 show that both trimethoprim and methylphenidate tend to dissolve in acidic NADES, which could be due to the presence of amine groups in these molecules (Figure 2). Trimethoprim has a pKa of 6.6, and a salt/cocrystal of methylphenidate with HCl has a pKa of 8.9 [39]; see Table 1. Ranitidine·HCl has some amine functions and is water soluble as salt, however, the water solubility rate depends on the crystal structure (polymorphism). This drug dissolves in all NADES tested. However, nitrofurantoin (with an NO_2_ group and some amide-functions, pKa of 7.2) [39] is not dissolved in any of the acidic NADES tested, but is only dissolved in CCAAProW (Table 2). At least the polarity of a NADES alone cannot predict the solubility of a compound [2,4,5].

### 2.3. Solubility and Stability of the Pharmaceuticals in Sugar-Based NADES

Table 4 presents the solubilities data of the pharmaceuticals in some sugar-based NADES. The sugar-based NADES dissolved only chloral hydrate, methylphenidate, and ranitidine∙HCl. Methylphenidate crystallized in glucose–fructose–water (GFW) a few days after solubilization. Griseofulvin, nitrofurantoin, spironolactone, and trimethoprim, are not soluble in any tested sugar-based NADES. The capability of three different groups of NADES to dissolve the pharmaceuticals is shown in a Venn diagram (Figure 3).

**Table 4:** Solubility of pharmaceuticals in the sugar-based NADES.

NADES	Molar Ratio	Pharmaceuticals						
		Ch	Gri	Mp	Nf	Ra	Spi	Tmp
G:F:W	1:1:10	++	−	+/P	−	++	−	−
S:F:W	1:1:10	++	−	++	−	++	−	−
S:F:G:W	1:1:1:11	++	−	+	−	++	−	−

The concentration of the tested pharmaceuticals was 5 mg/mL at 25 °C. The solubility was measured by visual inspection. ++: clear solution within 30 min by 30 min ultrasonication, +: clear solution in 48 h with ultrasonication, +/P: soluble within 30 min and precipitated within 24 h, (−): no solubility. F: d-fructose, G: d-glucose monohydrate, S: sucrose, W: water, Ch: chloral hydrate, Gri: griseofulvin, Nf: nitrofurantoin, Ra: ranitidine·HCl, Spi: spironolacton, Tmp: trimethoprim, and Mp: methylphenidate.

### 2.4. Solubility and Stability of the Pharmaceuticals in Selected NADES, Lactic Acid, Acetic Acid, and Propylene Glycol in a Range of Concentrations

The solubility and stability (at 4 °C) of the test drugs was studied in a range of concentrations in some selected NADES (Table 5). Two sugar-based NADES and some other NADES that previously showed to be able to dissolve most of the tested drugs, were chosen for this experiment. Not all drugs were tested on each of the NADES in this experiment because some of the drugs were previously found to not be soluble at all, or some dissolved easily in many NADES (like chloral hydrate and ranitidine∙HCl). The experiment focussed more on the poorly soluble drugs, such as methylphenidate, trimethoprim, griseofulvin, spironolactone, and nitrofurantoin. Vortexing, ultrasonication, and magnetic stirring were applied to dissolve the target compounds in the NADES. This took some minutes for lower concentrations (10–50 mg/mL) to several hours until a couple of days for higher concentrations (100–250 mg/mL), particularly in case of viscous NADES such as citric-acid–sucrose–water (CASW) and the sugar-based NADES. The results are shown in Table 5.

Chloral hydrate provided a transparent solution in the lactic-acid-based (zero-water containing) NADES such as LAF and LAPo, malic-acid-based- (MAFW and MAPoW), citric-acid-based- (CAPoW), and a sugar-based NADES (SFW), up to 250 mg/mL, which was the highest concentration tested. However, in CCAAProW, the maximum solubility was between 150 and 200 mg/mL.

Ranitidine·HCl was also soluble in the acid-based NADES and CCAAProW, up to 250 mg/mL, but it had a lower solubility in a sugar-based NADES of sucrose–glucose–fructose–water (SGFW), with a saturation concentration in the range of 150–200 mg/mL. The high concentration of ranitidine in the NADES gave it a brownish color.

Combining acid with propylene glycol seemed to improve the solubility of most drugs tested. Propylene glycol is a common excipient used in drug formulations. In drug delivery technology, polymers of lactic acid [poly(d,l-lactic acid)] and glycolide [poly(lactide-co-glycolide)] are used as carriers for both water-soluble and -insoluble drugs (microencapsulation/nanospheres/nanoparticles) [44,45]. A copolymer of poly (l-lactic acid) and poly (ethylene oxide) was used as a biodegradable hydrogel matrix for sustained-release drugs administered as subcutaneous injection [46], whereas a copolymer of poly (lactic acid)–poly(ethylene glycol)–poly(lactic acid) was used for preparation of nanospheres/microspheres of paclitaxel [47].

Since the combination of acetic acid or lactic acid with propylene glycol (1:1) (both liquids), dissolved most of the drugs tested, it was interesting to know whether the combination was necessary for solvation of the drugs, or instead each component itself could dissolve the drugs. Acetic acid is thought to be an excellent solvent for many organic compounds [39]. The solubility of the drugs was tested separately in acetic acid, lactic acid, or propylene glycol alone, at a concentration of 5–250 mg/mL; results are shown in Table 6.

**Figure 4:**
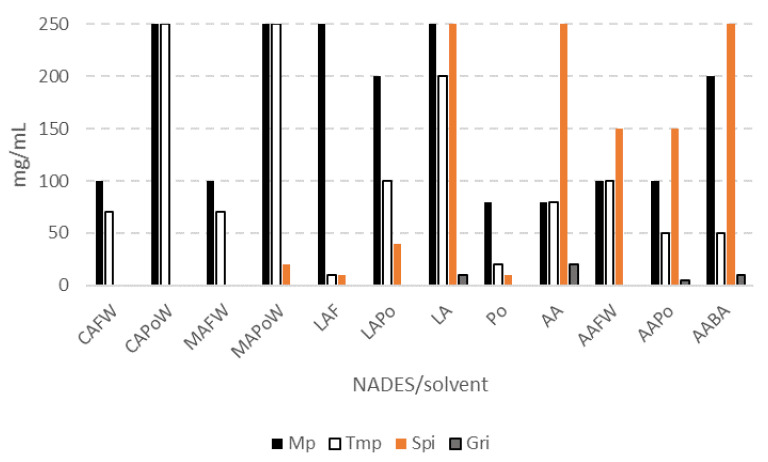
The capacity of some selected acid-based NADES, acetic acid (AA), lactic acid (LA), and propylene glycol (Po) to dissolve some non-water-soluble pharmaceuticals (mg/mL); methyl phenidate (Mp), trimethoprim (Tmp), spironolactone (Spi), and griseofulvin (Gri). Data extracted from Table 5 and Table 6. The stability test was observed for at least 4 months except those of Mp and Tmp in AAPo, AAFW, and AABA (1 month observation). BA: beta-alanine, CA: citric acid, MA: malic acid, F: fructose, and W: water.

Chloral hydrate and ranitidine·HCl are soluble in both acids and in propylene glycol alone, up till 250 mg/mL. Both compounds dissolved easily in almost all NADES tested. The solubility of the other non-water-soluble compounds (Methyl phenidate, trimethoprim, spironolactone, and griseofulvin) in some selected acid-based NADES are compared in Figure 4 (data extracted from Table 5 and Table 6).

Combination of citric acid or malic acid with propylene glycol provided the highest solubility of methylphenidate, as compared to those of acid–sugar combinations. The degree of capacity to dissolve methylphenidate among the citric-acid-based NADES was CAPoW > CAFW > CASW (250 > 100 > 10 mg/mL), which was also similar to the malic-acid-based NADES, MAPoW > MAFW (250 > 100 mg/mL). Interestingly, in the case of lactic acid, its combination with fructose (LAF) dissolved methylphenidate better than the combination with propylene glycol (LAPo), 250 vs. 200 mg/mL, respectively. In some acetic-acid-based NADES, the solubility of methylphenidate was in the order of AABA > AAFW = AAPo (200 > 100 and 100 mg/mL). Methylphenidate (pKa 8.9) was soluble in lactic acid up until 250 mg/mL; however, it dissolved in much lower amounts in acetic acid (50 mg/mL) and 80 mg/mL in a neutral component/solvent, such as propylene glycol. With a capacity to dissolve methylphenidate up until 250 mg/mL, the concentration achieved was 125-fold of the concentration found in commercial products (1–2 mg/mL). A lower solubility of this compound was observed in a sugar-based NADES (SFW, 20 mg/mL) and the ionic-liquid-DES of CCAAProW (10 mg/mL).

The degree of solubility of trimethoprim among the citric-acid-, malic-acid-, and lactic-acid-based NADES were found to be as follows, CAPoW > CAFW > CASW (250 > 70 > 20 mg/mL), MAPoW > MAFW (250 > 70 mg/mL), LAPo > LAF (100 > 10 mg/mL), and AAFW > AAPo = AABA (100 > 50 and 50 mg/mL). Trimethoprim was soluble in lactic acid up until 200 mg/mL, acetic acid (until 80 mg/mL) and propylene glycol (until 20 mg/mL).

Spironolacton, griseofulvin, and nitrofurantoin were the most challenging compounds in the tested group, with regards to the difficulty to be dissolved in NADES. Only some NADES that previously showed the solubility of the compounds (at 5 mg/mL), were chosen for this experiment. Spironolacton (practically insoluble in water) was soluble in a combination of acid and propylene glycol, with a degree of solubility as follows AAPo > LAPo > MAPo, (150 > 50 > 20 mg/mL). A combination of an acid with a sugar that could dissolve this compound, was only AAFW (150 mg/mL) and LAF (10 mg/mL). A combination of acetic acid and beta-alanine (AABA) without water could dissolve spironolactone up until 250 mg/mL, as well as in acetic acid or lactic acid alone. It also dissolved in propylene glycol, but with a much lower solubility (10 mg/mL). At the concentration of 250 mg/mL, the mixture of spironolactone and acetic acid or lactic acid provided a molar ratio of about 29:1 and 22:1, respectively. The lower solubility of spironolactone in propylene glycol might explain its lower solubility in some combinations of acid-propylene glycol (1:1); CAPoW, MAPoW, LAPo, AAPo (Table 5). Spironolactone, which has three ketone groups in its structure, most probably acts as an electron pair donor (“Lewis” base) towards compounds with a carboxyl group, like acetic acid, lactic acid, and beta-alanine, to form a hydrogen bond network. When the acid was combined with another neutral compound such as propylene glycol or fructose (as proton donor), the solubility of spironolactone decreased. The intermolecular interaction between spironolactone and an acid was most probably stronger than that formed by the alcohols.

Griseofulvin (practically insoluble in water) was only soluble in the combinations of acetic acid with propylene glycol (AAPo) or with beta-alanine (AABA), with the highest concentration of 5 mg/mL and 10 mg/mL, respectively. Griseofulvin was also better dissolved in acetic acid (20 mg/mL), or lactic acid (10 mg/mL) alone than in a mixture of acetic-acid–propylene-glycol (5 mg/mL). Whilst, in a combination of acetic-acid–beta-alanine (5:1), it was soluble at 10 mg/mL.

Nitrofurantoin was found soluble only in CCAAProW at 5 mg/mL. It did not dissolve in any of the acids tested, nor in propylene glycol at the lowest given concentration. Nevertheless, a supramolecular assembly of choline-chloride–acetic-acid–proline–water could dissolve nitrofurantoin at a concentration of 5 mg/mL, which was an improvement relative to its solubility in water (0.19 mg/mL) [39]. The combination of components in a NADES might not only improve a drug’s solubility, but might also be beneficial for the bioavailability.

## 3. Materials and Methods

### 3.1. Chemicals

All constituents of NADES used in this work were purchased from Sigma-Aldrich, St. Louis, MO, USA (purity > 99%), except d-Fructose (was obtained from Boom B.V., Meppel, The Netherlands), d-Glucose monohydrate (was purchased from Ducheva Biochemie, Haarlem, The Netherlands) and glycerol (was provided from Merck Schuchardt, Hohenbrunn, Germany).

The tested pharmaceuticals (trichloroacetaldehyde monohydrate or chloral hydrate, griseofulvin, methylphenidate, nitrofurantoin, ranitidine·HCl, spironolacton, trimethoprim) were provided by Tiofarma, Oud-Beijerland, The Netherlands.

### 3.2. Preparation of Deep Eutectic Solvents

A NADES is composed by two or more components and water (if necessary) in a certain molar ratio. Based on the required molar ratio, the components and water were weighed and mixed in a glass bottle. The mixture was homogenized by placing the bottle on a magnetic stirrer plate, at e.g., 700–900 rpm, at room temperature or with heating at 50 °C. A transparent mixture was formed after homogenization, which took from 1–24 h, depending on the NADES composition. If a less/minimum content of water in the NADES was needed, the mixture could be placed in a freeze-dryer for 24–48 h (the total weight of the mixture before and after freeze drying were used to measure the water lost/remaining in the mixture). NADES obtained in this way contained water as one of the constituents, whereas a zero-water containing NADES could be made by mixing/ melting the non-water ingredients together (with a heating at 50–80 °C if necessary), under continuous stirring. A stable NADES remains transparent and shows no aggregation/crystallization or any microbial contamination, after a long period of storage (at least >12 months’ observation) at room temperature.

### 3.3. Solubility and Stability Test

One milliliter of each NADES was transferred into a 10 mL glass vial. Five milligram sample of a pharmaceutical was mixed with the NADES in the vial and vortexed for a few seconds. The mixture was further ultrasonicated for 30 min and stirred on a magnetic stirrer-plate. Solubility was measured by visual inspection at certain time-points for 48 h. NADES that visually resulted in full dissolution of the test compound were subsequently observed for their capabilities to dissolve the test-compound in a range of concentrations (10–250 mg/mL), including their stability. For a relatively high concentration of compound (100–250 mg/mL), the solubilization process might take a couple of days (with some hours of magnetic stirring per day). The stability of the NADES solution was tested up until four months at 4 °C.

## 4. Conclusions

Considering the aim of this study, one could draw several conclusions. First, by screening by hand and visual inspection of the vials with the NADES and target compounds for all tested medicines, a solvent was found that gave a manifold increase of the solubility of the target compound if compared to an aqueous solution.

Moreover, it seems that NADES are highly selective solvents, for every compound, one needs to optimize the solvent, both in terms of the components, their molar ratios, and their water content. Some drugs dissolved easily in the highest amount tested. It seemed that in this case, the compounds might have become part of a new NADES. Considering structural features of the tested drugs, it seems that compounds with amine functions (such as methylphenidate and trimethoprim) have good solubility in acid-containing NADES. Spironolacton and griseofulvin dissolve better in acetic acid and lactic acid alone, than in a mixture of each of these acids with a neutral compound such as propylene glycol or fructose. The solubility of spironolactone and griseofulvin in propylene glycol is lower than in any acids. A stable mixture of spironolactone with acetic acid or lactic acid with a molar ratio of about 29:1 or 22:1, respectively, could be achieved, which could be considered as DES/NADES. The only NADES that could dissolve nitrofurantoin was choline-chloride–acetic-acid–proline–water (1:1:1:5) at a concentration of 5 mg/mL. All solutions/mixtures tested were stable and transparent (observed visually) at 4 °C storage, for at least 4 months.

Thus, NADES are promising solvents for liquid formulations of pharmaceuticals, or might be formulated as part of a deep eutectic mixture with non-toxic common natural compounds. For application of NADES in drug formulations, the pharmacokinetics, and particularly the bioavailability of the drugs in NADES, need to be studied. The use of NADES for parenteral and tube-feeding needs further development. Moreover, in-depth studies of the various systems of NADES constituents and the solutes are needed to get more insight into the role of the various variables in the systems. Various spectroscopies should help to get insight into the physico-chemical process of dissolution of compounds with ILs and NADES.

## Figures and Tables

**Table 1:** Solubilities and pKa (s) of the APIs and NADES components [39].

Compound	pKa/PH	Solubility (in g/mL), Uses, and Some Other Information
Chloral Hydrate (Ch)	-	Freely soluble in water (2.4, 0 °C), (8.3, 25 °C), (14.3, 40 °C), but undergoes dissociation. Soluble in ethanol (0.77), chloroform (0.50), ether (0.66), glycerol (2.0), and olive oil (0.71). Freely soluble in acetone, methyl ethyl ketone. Moderately or sparingly soluble in turpentine, petroleum ether, carbon tetrachloride, benzene, and toluene. Therapeutic use—sedative
Griseofulvin (Gri)	-	Practically insoluble in water, petroleum ether. Slightly soluble in ethanol, methanol, acetone, benzene, chloroform, ethyl acetate, and acetic acid. Soluble in DMF (0.12–0.14). Therapeutic use—antifungal
Methylphenidate (Mp)	pKa 8.9 (of HCl crystals)	Practically insoluble in water and petroleum ether. Soluble in ethanol, ethyl acetate, and ether. Methylphenidate∙HCl is soluble in water, ethanol, and chloroform. Therapeutic use—CNS stimulant
Nitrofurantoin (Nf)	pKa 7.2	Soluble in water pH 7 (0.00019), 95% ethanol (0.00051), acetone (0.0051), DMF (0.08), peanut oil (0.000021), glycerol (0.0006), and polyethylene glycol (0.015). Therapeutic use—antibacterial
Ranitidine∙HCl (Ra)	-	Freely soluble in water and acetic acid. Soluble in methanol and sparingly soluble in ethanol. Practically insoluble in chloroform. Therapeutic use—antiulcer
Spironolactone (Spi)	-	Soluble in most organic solvents and practically insoluble in water. Therapeutic use—diuretic
Trimethoprim (Tmp)	pKa 6.6	Soluble (at 25 °C) in DMAC (0.139), benzyl alcohol (0.073), propylene glycol (0.026), chloroform (0.018), methanol (0.012), water (0.0004), ether (0.00003), And benzene (0.00002). Therapeutic use—antibacterial
Choline chloride (CC)	-	Very soluble in water (neutral) and ethanol.
Betaine (Be)	-	Soluble in water (1.60), methanol (0.43), and ethanol (0.07). Sparingly soluble in ether. The pH of saturated solution of betaine monohydrate is about 8.0. Solubility of Betaine∙HCl (at 25 °C) in water, 0.65; ethanol, 0.04. Practically insoluble in chloroform and ether. The pH of 5% (*w*/*v*) aqueous solution = 1.0. Therapeutic use—in treatment of homocystinuria
Beta-Alanine (BA)	pK_1_ 3.60 pK_2_ 10.19	Freely soluble in water (pH of 5% aqueous solution: 6.0–7.3). Slightly soluble in ethanol; practically insoluble in ether and acetone.
l-Proline (Pro)	pI 6.30 pK_1_ 1.99 pK_2_ 10.60	Soluble in water (1.27, 0 °C), (1.62, 25 °C), (2.07, 50 °C), (2.39, 65 °C), and ethanol (0.012, 35 °C). Insoluble in ether, butanol, and isopropanol.
Acetic acid (AA)	pKa 4.74	An excellent solvent for many organic compounds. Miscible with water, ethanol, glycerol, ether, and carbon tetrachloride. pH of aqueous solution—2.4 (1.0 M); 2.9 (0.1 M); 3.4 (0.01 M). Used in pharmaceutical and food industries as acidifier and preservative. LD_50_ orally in rats—3.73 g/kg.
Lactic acid (LA)	pKa 3.86	Soluble in water, ethanol, furfurol; less soluble in ether. Practically insoluble in chloroform, petroleum ether, and carbon disulfide. There are a wide range of applications of LA in pharmaceutical-, food- and cosmetics industries. LD_50_ orally in rats—3.73 g/kg.
dl-Malic acid (MA)	-	Solubility (at 20 °C) in: water, 0.56; methanol, 0.65; ethanol, 0.36; acetone, 0.14; dioxane, 0.23; and diethyl ether, 0.006. Practically insoluble in benzene. Used in pharmaceutical and food industries as flavoring agent, flavor enhancer, and acidulant.
Citric acid (CA)	pK_1_ 3.128 pK_2_ 4.761 pK_3_ 6.396	Solubility of the anhydrate form in water increases with higher temperatures, e.g., 0.59 at 20 °C; 0.71 at 50 °C; and 0.84 at 100 °C. pH of 0.1 N solution = 2.2. Solubility of the monohydrate crystals in ether, 0.015; chloroform, 0.0001; amyl alcohol, 0.125; amyl acetate, 0.052; and ethyl acetate, 0.048; methanol, 1.56 (at 19 °C); propanol, 0.49 (at 19 °C). Pharmaceutical incompatibilities—K-tartrate, alkali- and alkaline earth carbonates and bicarbonates, acetates, sulfides. Used in pharmaceutical, food and beverage industries as acidulant, effervescent, pH adjuster, antioxidant. LD_50_ i.p. in rats—975 mg/kg.
Propylene glycol (Po)	-	Hygroscopic, viscous liquid. Miscible with water, acetone, chloroform. Soluble in ether; dissolves essential oils; and immiscible with fixed oils. It’s stable at room temperature, but tends to oxidize at high temperatures to produce propionaldehyde, lactic acid, pyruvic acid, and acetic acid. Used in pharmaceutical and food industries as solvent, emulsifier, and humectant. LD_50_ orally in rats—25 mL/kg.
Glycerol (Go)	-	Hygroscopic syrupy liquid with sweet warm taste (about 0.6 times as sweet as cane sugar), neutral to litmus. Miscible with water, ethanol. Soluble in ethyl acetate (1:11) and ethyl ether (1:500). Insoluble in benzene, chloroform, carbon tetrachloride, carbon disulfide, petroleum ether, and oils. Used in pharmaceutical, food & cosmetics industries as solvent, humectant, emollient, and sweetener. LD_50_ in rats—orally, >20 mL/kg; i.v. 4.4 mL.
Xylitol (Xo)	-	Solubility of the stable form (orthorhombic needles or prisms) in methanol, 0.047; ethanol, 0.0095; and water, 0.642. Use: as oral nutrient (sweetness equal to sucrose), i.v. nutrient, and in anticaries preparation. LD_50_ orally in mice: approx. 22 g/kg.
Sorbitol (So)	pH 7.0	Freely soluble in water (up to 0.83), a concentrated solution provides a higher viscosity than its corresponding glycerol solution. Sparingly soluble in cold ethanol, but solubility increases with increased temperature. Soluble in methanol, isopropanol, butanol, cyclohexanol, phenol, acetone, acetic acid, DMF, pyridine, and acet-amide solutions. Uses in food industries—sugar substitute for diabetics, humectant, softener in peanut/coconut butter, sequestrant in soft drinks and wines, to reduce undesirable aftertaste of saccharin. Uses in pharmaceutical industry—flavor agent, excipient in tablet to increase absorption of vitamins, and laxative.
Mannitol (Mo)	pKa 13.5 (18 °C)	Soluble (at 25 °C) in water (0.18), ethanol (0.012), and glycerol (0.055). More soluble in hot water. Soluble in pyridine, aniline, aqueous solution of alkalis. Insoluble in ether. Used in pharmaceutical and food industries as excipient, diluent, lubricant, anticaking agent, stabilizer, thickener, sweetener, and flavoring agent. Used in therapy as diuretic and diagnostic aid of renal function.
d-Fructose (F)	pKa 12.06 (18 °C)	Occurs in both furanose and pyranose forms. Freely soluble in water. Soluble in ethanol (0.066) and methanol (0.071). Slightly soluble in cold, but freely soluble in hot acetone. Soluble in pyridine, ethylamine, and methylamine.
α-d-Glucose (G)	pH 5.9 (0.5 M aqueous solution)	The α-form-monohydrate is soluble in water (1.0) and ethanol (0.017). The α-form-anhydrate is soluble in water (0.91, 25 °C); (1.25, 30 °C); (2.44, 50 °C); (3.57, 70 °C); (5.55, 90 °C), and in methanol (0.008, 20 °C). Very sparingly soluble in ethanol, ether, and acetone. Soluble in hot glacial acetic acid, pyridine, and aniline.

**Table 2:** Solubility of pharmaceuticals in the choline chloride- or betaine-based NADES.

NADES	Molar Ratio	Pharmaceuticals						
		Ch	Gri	Mp	Nf	Ra	Spi	Tmp
CC:LA	1:1	++	−	++	−	++	−	−
CC:MA:W	1:1:4	++	−	++	−	++	−	−
CC:MeA:W	1:1:4	++	−	++	−	++	−	−
CC:CA:W	1:1:6	++	−	++	−	++	−	−
CC:Po:W	1:1:1	++	−	++	−	++	−	−
CC:Go:W	1:1:1	++	−	+/P	−	+	−	−
CC:So:W	3:1:6	++	−	−	−	+	−	−
CC:G:W	5:2:5	++	−	−	−	+	−	−
CC:F:W	1:1:3	++	−	−	−	+	−	−
CC:S:W	4:1:7	++	−	−	−	−	−	−
CC:Man:W	5:2:5	++	−	−	−	+	−	−
CC:Tre:W	4:1:5	++	−	−	−	+	−	−
CC:X:W	2:1:2	++	−	−	−	+	−	−
CC:MA:Xo:W	1:1:1:4	++	−	+/P	−	++	−	−
CC:MA:Pro:W	1:1:1:4	++	−	+/P	−	++	−	−
CC:AA:Pro:W	1:1:1:5	++	−	++	+	++	+/P	−
Be:S:W	2:1:8	++	−	++	−	++	−	−
Be:MA:W	1:1:7	++	−	++	−	++	−	++
Be:MA:G:W	1:1:1:7	++	−	+/P	−	++	−	−
Be:MA:Pro:W	1:1:1:7	++	−	++	−	++	−	++
Be:AA:Pro:W	1:1:1:5	+/P	−	+/P	−	+/P	−	−

The concentration of the tested pharmaceuticals was 5 mg/mL at 25 °C. The solubility was measured by visual inspection. ++: clear solution within 30 min by vortexing and 25 min ultrasonication, +: clear solution in 24 h with vortexing, ultrasonication, and magnetic stirring, +/P: soluble within 30 min and precipitated within 24 h, (−): no solubility. CC: choline-chloride, Be: betaine, AA: acetic acid, LA: lactic acid, MeA: maleic acid, MA: dl-malic acid, CA: citric acid monohydrate, Go: glycerol, Po: propylene glycol, Xo: xylitol, So: d-sorbitol, X: d-xylose, G: d-glucose monohydrate, F: d-fructose, Man: d-mannose, Gal: d-galactose, S: sucrose, Tre: d-trehalose dihydrate, Pro: l-proline, W: water, Ch: chloral hydrate, Gri: griseofulvin, Nf: nitrofurantoin, Ra: ranitidine·HCl, Spi: spironolacton, Tmp: trimethoprim, and Mp: methylphenidate.

**Table 5:** Solubility and stability of the pharmaceuticals in a range of concentrations (10–250 mg/mL) in the selected NADES.

NADES	Molar Ratio of NADES Components	wt.% of Water	Drug Tested	Observation of Solubility of Drug (mg/mL) in NADES									Molar Ratio of NADES and Drug ^(a)^
				10	20	30	50	70	100	150	200	250	
CC:AA:Pro:W	1:1:1:5	22	Ch	+	+	+	+	+	+	+	+/P	+/P	3:3:3:15:1
			Ra	+	+	+	+	+	+	+	+	+	4:4:4:20:1
			Mp	+	−	np	np	np	np	np	np	np	np
			Nf	+P	−	np	np	np	np	np	np	np	np
CA:F:W	1:1:5	19	Mp	+	+	+	+	+	+	−	np	np	6:6:38:1
			Tmp	+	+	+	+	+	−	np	np	np	11:11:68:1
CA:S:W	1:1:6	16	Mp	+	−	np	np	np	np	np	np	np	np
			Tmp	+	+	−	np	np	np	np	np	np	np
CA:Po:W	1:1:4	21	Ch	+	+	+	+	+	+	+	+	+	5:5:20:2
			Ra	+	+	+	+	+	+	+	+	+	5:5:21:1
			Mp	+	+	+	+	+	+	+	+	+	7:7:28:2
			Tmp	+	+	+	+	+	+	+	+	+	4:4:17.5:1
			Spi	−	−	np	np	np	np	np	np	np	np
MA:F:W	1:1:7	28	Ch	+	+	+	+	+	+	+	+	+	2:2:14:1
			Ra	+	+	+	+	+	+	+	+	+	4:4:29:1
			Mp	+	+	+	+	+	+	+/P	np	np	7:7:52:1
			Tmp	+	+	+	+	+	+/P	np	np	np	13:13:93:1
MA:Po:W	1:1:3	20	Ch	+	+	+	+	+	+	+	+	+	3:3:9:1
			Ra	+	+	+	+	+	+	+	+	+	6:6:19:1
			Mp	+	+	+	+	+	+	+	+	+	4:4:13:1
			Tmp	+	+	+	+	+	+	+	+	+	5:5:16:1
			Spi	+	+	−	np	np	np	np	np	np	np
LA:F	5:1	0	Ch	+	+	+	+	+	+	+	+	+	6:1:1
			Ra	+	+	+	+	+	+	+	+	+	13:3:1
			Mp	+	+	+	+	+	+	+	+	+	9:2:1
			Tmp	+	−	np	np	np	np	np	np	np	np
			Spi	+	−	np	np	np	np	np	np	np	np
LA:Po	1:1	0	Ch	+	+	+	+	+	+	+	+	+	9:9:2
			Ra	+	+	+	+	+	+	+	+	+	10:10:1
			Mp	+	+	+	+	+	+	+	+	−	8:8:1
			Tmp	+	+	+	+	+	+	−	np	np	19:19:1
			Spi	+	+	+	+	−	np	np	np	np	56:56:1
AA:F:W	5:2:5	27	Mp	+	+	+	+	+	+	+/P	np	np	19:8:19:1
			Tmp	+	+	+	+	+	+	+/P	np	np	24:10:24:1
			Spi	+	+	+	+	+	+	+	+/P	np	23:9:23:1
AA:Po	1:1	0	Mp	+	+	+	+	+	+	+/P	np	np	17:17:1
			Tmp	+	+	+	+	+/P	np	np	np	np	42:42:1
			Spi	+	+	+	+	+	+	+	+/P	+/P	20:20:1
			Gri	+/P	−	np	np	np	np	np	np	np	np
AA:BA	5:1	0	Mp	+	+	+	+	+	+	+	+	+/P	17:3:1
			Tmp	+	+	+	+	+/P	np	np	np	np	85:17:1
			Spi	+	+	+	+	+	+	+	+	+	24:5:1
			Gri	+	−	np	np	np	np	np	np	np	np
S:F:W	1:1:10	25	Ch	+	+	+	+	+	+	+	+	+	4:4:40:3
			Ra	+	+	+	+	+	+	+	+	+	3:3:28:1
			Mp	+	+	−	np	np	np	np	np	np	np
S:F:G:W	1:1:1:11	22	Ra	+	+	+	+	+	+	+	−	np	4:4:4:44:1
			Tmp	−	Np	np	np	np	np	np	np	np	np

+: clear solution for four months at room temperature or 4 °C, +/P: soluble but precipitated after 24 h, (−): not soluble, np: not performed. ^(a)^ Molar ratio of NADES components and test-drug at the highest drug’s concentration observed (component 1: component 2: component 3: water: drug). AA: acetic acid, BA: beta-Alanine, CC: choline-chloride, CA: citric acid monohydrate, MA: malic acid, LA: l-lactic acid, F: d-fructose, G: d-glucose monohydrate, S: sucrose, Po: propylene glycol, Pro: l-proline, W: water, Ch: chloral hydrate, Gri: griseofulvin, Mp: methylphenidate, Nf: nitrofurantoin, Ra: ranitidine·HCl, Spi: spironolacton, and Tmp: trimethoprim.

**Table 6:** Solubility test of pharmaceuticals in acetic acid, l-lactic acid, or propylene glycol.

Solvent	Drug Tested	Solubility (mg/mL)									Molar Ratio ^(a)^ Solvent:Drug
		5	10	20	50	80	100	150	200	250	
Acetic acid	Ch	+	+	+	+	+	+	+	+	+	11, 6:1
	Ra	+	+	+	+	+	+	+	+	+	24, 5:1
	Mp	+	+	+	+	+	+/P	np	np	np	51:1
	Tmp	+	+	+	+	+	+/P	+/P	np	np	63, 4:1
	Spi	+	+	+	+	+	+	+	+	+	29:1
	Gri	+	+	+	+/P	+/P	np	np	np	np	np
	Nf	−	−	np	np	np	np	np	np	np	np
l−Lactic acid	Ch	+	+	+	+	+	+	+	+	+	9:1
	Ra	+	+	+	+	+	+	+	+	+	18, 8:1
	Mp	+	+	+	+	+	+	+	+	+	12, 5:1
	Tmp	+	+	+	+	+	+	+	+	−	19, 4:1
	Spi	+	+	+	+	+	+	+	+	+	22:1
	Gri	+	+	−	np	np	np	np	np	np	np
	Nf	−	−	np	np	np	np	np	np	np	np
Propylene glycol	Ch	+	+	+	+	+	+	+	+	+	9:1
	Ra	+	+	+	+	+	+	+	+	+	19:1
	Mp	+	+	+	+	+	−	np	np	np	40:1
	Tmp	+	+	+	−	np	np	np	np	np	198:1
	Spi	+	+	−	np	np	np	np	np	np	569:1
	Gri	−	−	np	np	np	np	np	np	np	np
	Nf	−	−	np	np	np	np	np	np	np	np

+: clear solution within 30 min, +/P: soluble, but crystallized/precipitated after 24 h, −: not- or partly soluble, np: not performed. ^(a)^ Molar ratio of solvent (acetic acid, lactic acid or propylene glycol) and test-drug at the highest drug’s concentration observed (solvent: drug). Ch: chloral hydrate, Gri: griseofulvin, Mp: methylphenidate, Nf: nitrofurantoin, Ra: ranitidine·HCl, Spi: spironolacton, and Tmp: trimethoprim.

## Sample Availability

The tested pharmaceuticals (trichloroacetaldehyde monohydrate or chloral hydrate, griseofulvin, methylphenidate, nitrofurantoin, ranitidine·HCl, spironolacton, trime-thoprim) were provided by Tiofarma, Oud-Beijerland, The Netherlands (V.S.S.).
